# Frequency of prothrombin time-international normalized ratio monitoring and the long-term prognosis in patients with mechanical valve replacement

**DOI:** 10.1186/s12872-023-03293-w

**Published:** 2023-06-24

**Authors:** Le Geng, Jiaxi Gu, Minghui Li, Hong Liu, Haoliang Sun, Buqing Ni, Weidong Gu, Yongfeng Shao, Mingfang Li, Minglong Chen

**Affiliations:** 1 Division of Cardiovascular Surgery, the First Affiliated Hospital of Nanjing Medical University, 300 Guangzhou Road, Nanjing, 210029 P.R. China; 2 Division of Cardiology, the First Affiliated Hospital of Nanjing Medical University, 300 Guangzhou Road, Nanjing, 210029 P.R. China

**Keywords:** Mechanical heart valve, Warfarin, Prothrombin time-international normalized ratio, Monitoring frequency

## Abstract

**Background:**

The study aimed to assess the correlation between the monitoring frequency of PT-INR and the long-term prognosis in patients with mechanical heart valve (MHV) replacement after discharge.

**Methods:**

This single-center, observational study enrolled patients who underwent MHV replacement and discharged from June 2015 to May 2018. Patients or their corresponding family members were followed with a telephone questionnaire survey in July-October 2020. Based on monitoring intervals, patients were divided into frequent monitoring (FM) group (≤ 1 month) and less frequent monitoring (LFM) group (> 1 month). The primary endpoint was the composite of thromboembolic event, major bleeding or all-cause death. The secondary endpoints were thromboembolic event, major bleeding or all-cause death, respectively.

**Results:**

A total of 188 patients were included in the final analysis. The median follow-up duration was 3.6 years (Interquartile range: 2.6 to 4.4 years). 104 (55.3%) patients and 84 (44.7%) patients were classified into the FM group and the LFM group, respectively. The FM group had a significantly lower incidence of the primary endpoint than the LFM group (3.74 vs. 1.16 per 100 patient-years, adjusted HR: 3.31 [95% CI 1.05–10.42, P = 0.041]). Secondary analysis revealed that the risk of thromboembolic events and all-cause death were also reduced in the FM group.

**Conclusions:**

The management of warfarin treatment in patients after MHV replacement remains challenging. Patients with less frequent monitoring of PT-INR might have worse clinical prognosis than those with frequent PT-INR monitoring.

**Supplementary Information:**

The online version contains supplementary material available at 10.1186/s12872-023-03293-w.

## Background

Valvular heart disease (VHD) can lead to heart failure, stroke and even death if left untreated [1, 2]. VHD is one of the most common heart diseases in China, with a weighted prevalence of 3.8% [3]. Rheumatic infection is the main cause of VHD in China [3, 4]. Although the incidence of rheumatic VHD decreases gradually, the number of patients with VHD remains large due to the large and aging population in China.

Prosthetic valve replacement is an important and reliable treatment for patients with VHD [5, 6]. Bioprosthetic heart valves (BHVs) are subject to structural valve deterioration, especially in young adult patients, while mechanical heart valves (MHVs) are more durable and can last lifelong. Therefore, MHVs are widely used in patients younger than 60 years [7, 8]. However, patients should require lifelong anticoagulation with vitamin K antagonists after MHV replacement due to the high risk of thromboembolism caused by MHV [9, 10]. Warfarin is the most commonly prescribed vitamin K antagonist in China. It has several significant disadvantages including its narrow therapeutic window, potential drug-drug and drug-food interactions, unpredictable dose-response relationship, and the requirement of regular blood monitoring [11, 12]. Improper use of warfarin may cause thromboembolic or hemorrhagic complications, which are frequently fatal or result in permanent disability. Considering patients receiving MHV replacement are relatively young, these complications are most likely catastrophic to patients and increase the financial and emotional burden of their families and society. Therefore, ensuring the quality of warfarin therapy is crucial. To find an optimal balance between efficacy and safety, continuous blood monitoring is required to ensure the value of prothrombin time-the international normalized ratio (PT-INR) is within the therapeutic range [13]. In addition, the time in therapeutic range (TTR) is often calculated to assess the quality of anticoagulation therapy [14].

However, little is known about the quality of warfarin therapy after MHV replacement in China. Herein, we proposed the monitoring frequency of PT-INR as an index of patient compliance and hypothesized that the monitoring frequency of PT-INR was correlated with the long-term prognosis in patients after MHV replacement.

## Methods

### Study design

This single-center, observational study was approved by the medical ethics committee of the First Affiliated Hospital of Nanjing Medical University and conducted according to the Declaration of Helsinki and institutional guidelines.

### Study population

We included patients who underwent MHV replacement and discharged from our institution between June 2015 and May 2018. The patients or their family members who could not be contacted via phone or declined to answer telephone questionnaires were excluded from this study. A retrospective chart review was performed for all patients. Demographic data, clinical information before the MHV replacement, and procedure-related details were collected.

### Questionnaire

Between July 2020 and October 2020, a telephone follow-up questionnaire survey to patients or their family members was designed and conducted to evaluate the clinical outcomes after MHV replacement. The questionnaire contained 10 questions about the frequency of PT-INR monitoring and the occurrence of all-cause death, thromboembolic events and major bleeding (Additional File). It took 10 min or less to complete the telephone interview for all subjects. If the patients or their family members could not be reached with the initial telephone call, at least 3 additional attempts would be made. All calls were made by a single study coordinator (S.M.). During each phone call, S.M. explained the purpose of this questionnaire survey. Verbal informed consent was obtained from all patients or their family members. In order to minimize potential bias in the administration of the questionnaire and the responses from the patients or their family members, the telephone interview was based on a pre-written script.

### Outcomes

The primary endpoint was the composite of thromboembolic event, major bleeding or all-cause death. The secondary endpoints were thromboembolic event, major bleeding or all-cause death, respectively.

### Definitions

According to the criteria of the International Society on Thrombosis and Hemostasis (ISTH), major bleeding was defined as bleeding leading to a decrease in the hemoglobin level of at least 2 g/dl, transfusion of at least 2 units of packed red cells, occurring at a critical site, or resulting in death [15]. Based on the frequency of PT-INR monitoring, patients were classified into 2 major groups: frequent monitoring (FM) group with a monitoring interval ≤ 1 month, and less frequent monitoring (LFM) group with a monitoring interval > 1 month.

### Statistical analysis

All of the continuous variables investigated in our study were normally distributed and presented as mean with standard deviations. Differences between groups were tested using the Student’s *t* test. The categorical variables were expressed as counts with percentages and compared using the Chi-Squared test or Fisher’s exact test, as appropriate. A multivariable Cox proportional hazards model was conducted from the clinical outcome measure by frequent/less frequent INR monitoring. The variables including age and gender were adjusted. Hazard ratios (HRs) and their corresponding 95% confidence intervals (CIs) were used to estimate the effect size. A *P* value < 0.05 was considered significantly different. The software package SPSS version 26.0 (IBM, Cooperation, New York, NY, USA) was used for statistical analysis.

## Results

**Fig. 1:**
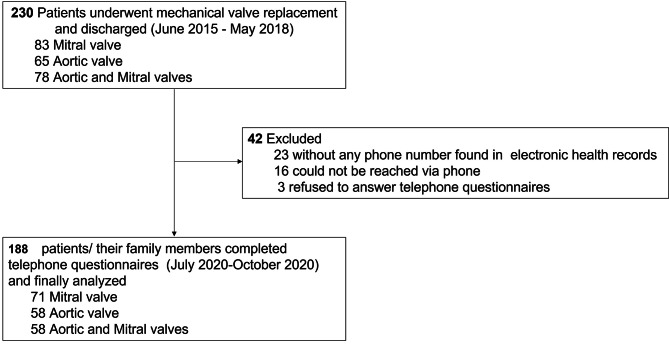
Flow of the study. CONSORT flow diagram. Between June 2015 to May 2018, 230 patients who underwent MHV replacement were discharged from our institution, of whom 188 were finally analyzed. Overall, 42 patients lost of follow-up were excluded from this study due to the following reasons: 23 patients had no telephone number found in electronic health records; 16 patients could not be reached via phone; 3 patients declined to answer telephone questionnaires

**Fig. 2:**
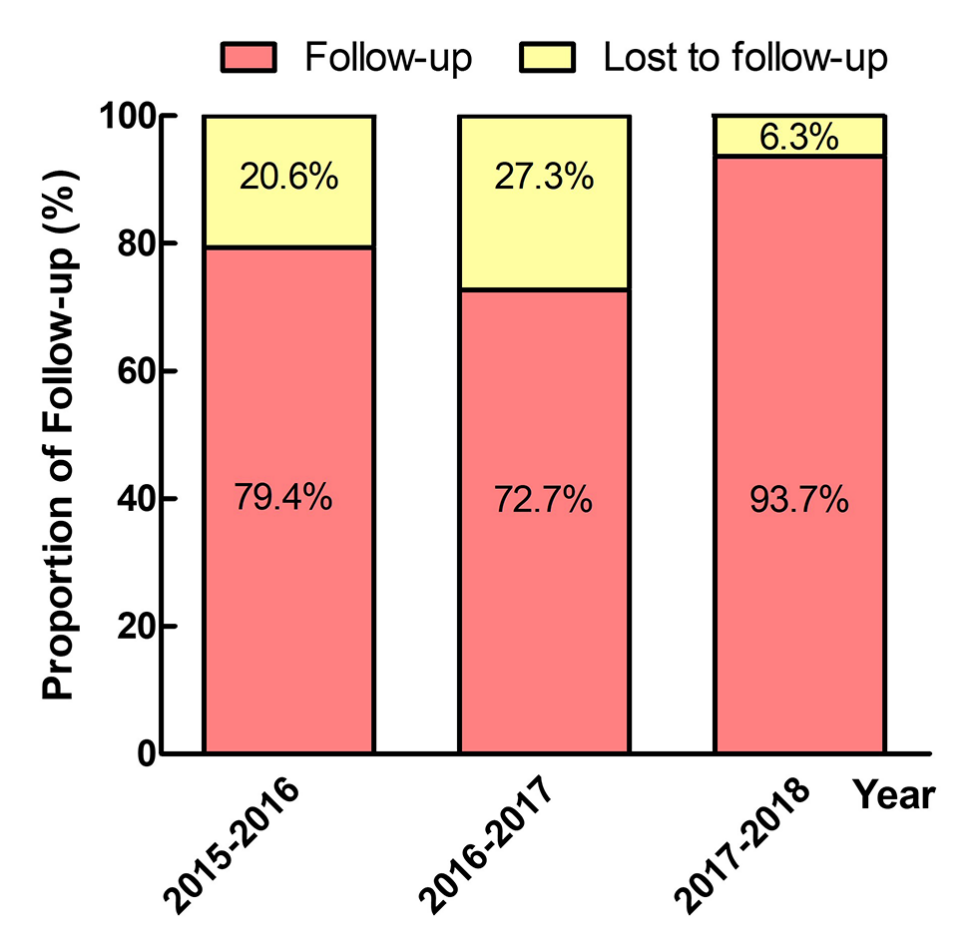
Proportion of lost to follow-up based on the year of mechanical heart valve replacement. The proportion of lost to follow-up was 20.6%, 27.3% and 6.3%, respectively based on the year of mechanical heart valve replacement from 2015 to 2018, with a significant decreasing trend (*P* < 0.01)

The flow of this study is shown in Fig. 1. Between June 2015 to May 2018, 230 patients who underwent MHV replacement were discharged from our institution, of whom 188 were analyzed. Overall, 42 patients were lost of follow-up and excluded from this study due to the following reasons: 23 patients had no telephone number recorded in their electronic health records; 16 patients could not be reached; 3 patients declined to answer the telephone questionnaire. As Fig. 2 shows, the proportion of lost to follow-up patients was 20.6%, 27.3% and 6.3%, respectively based on the year of MHV replacement from 2015 to 2018, with a significant decreasing trend (*P* < 0.01).

### Baseline characteristics

Of the 188 patients enrolled in this study, the mean age was 47.7 ± 7.9 years, and 94 (50%) patients were men. Regarding the surgical procedure, 71 patients (37.8%) underwent mechanical mitral valve replacement, 58 patients (30.8%) underwent mechanical aortic valve replacement, and 58 patients (30.8%) underwent both mechanical mitral and aortic valve replacement. The baseline characteristics of the 188 study subjects are shown in Table 1. Based on the PT-INR monitoring intervals, 104 patients were classified into the FM group, and another 84 patients were classified into the LFM group. Patients in the LFM group were less likely to have diabetes (5.8% vs. 0%, *P* = 0.03), and they had a larger left ventricular diastolic diameter (57.0 ± 11.0 mm vs. 53.7 ± 9.4 mm, *P* = 0.03) and smaller left ventricular ejection fraction (59.0 ± 7.55% vs. 61.6 ± 6.5%, *P* = 0.01). Patients who were lost to follow-up were more likely to have rheumatic heart disease (69% vs. 46%, *P* = 0.01) and less likely to have hypertension (5% vs. 22%, *P* = 0.01) than those with follow-up. No other significant differences in baseline characteristics between the patients with follow-up and patients who were lost to follow-up were found (Additional File Table 1).

**Table 1:** Baseline Characteristics of the Patients in FM and LFM Group

Variable	FM Group (n = 104)	LFM Group (n = 84)	P
**Demographic**			
Male (n, %)	47 (45.2%)	47 (56.0%)	0.19
Age (y)	47.1 ± 8.0	48.3 ± 7.7	0.28
**Disease history**			
Hypertension (n, %)	24 (23.1%)	18 (21.4%)	0.86
Diabetes (n, %)	6 (5.8%)	0 (0%)	0.03
Coronary artery disease (n, %)	7 (6.7%)	4 (4.8%)	0.76
Atrial fibrillation (n, %)	46 (44.2%)	39 (46.4%)	0.76
Ischemic stroke (n, %)	12 (11.5%)	12 (14.3%)	0.58
Creatine clearance (mL/min)	104.7 ± 37.6	99.8 ± 25.6	0.35
**Cardiac Echo parameter**			
Left atrial diameter (mm)	47.4 ± 10.1	46.6 ± 10.5	0.58
Left ventricular diastolic diameter (mm)	53.7 ± 9.4	57.0 ± 11.0	0.03
Left ventricular ejection fraction (%)	61.6 ± 6.5	59.0 ± 7.5	0.01
Left atrial embolus, n (%)	11 (10.6%)	9 (10.7%)	0.98
Rheumatic cause (n, %)	49 (47.1%)	37 (44.0%)	0.77
**Surgery information**			
Mitral valve alone (n, %)	42 (40.4%)	29 (34.5%)	0.45
Aortic valve alone (n, %)	29 (27.9%)	29 (34.5%)	0.35
Mitral valve + Aortic valve (n, %)	32 (30.8%)	26 (31.0%)	0.99
Concomitant atrial fibrillation ablation (n, %)	41 (39.4%)	33 (39.3%)	0.98
Concomitant coronary artery bypass grafting (n, %)	4 (3.8%)	5 (6.0%)	0.52
Concomitant anti-platelet drugs (n, %)	4 (3.8%)	5 (6.0%)	0.52

*FM*: frequent monitoring; *LFM*: less frequent monitoring

### Outcomes

**Fig. 3:**
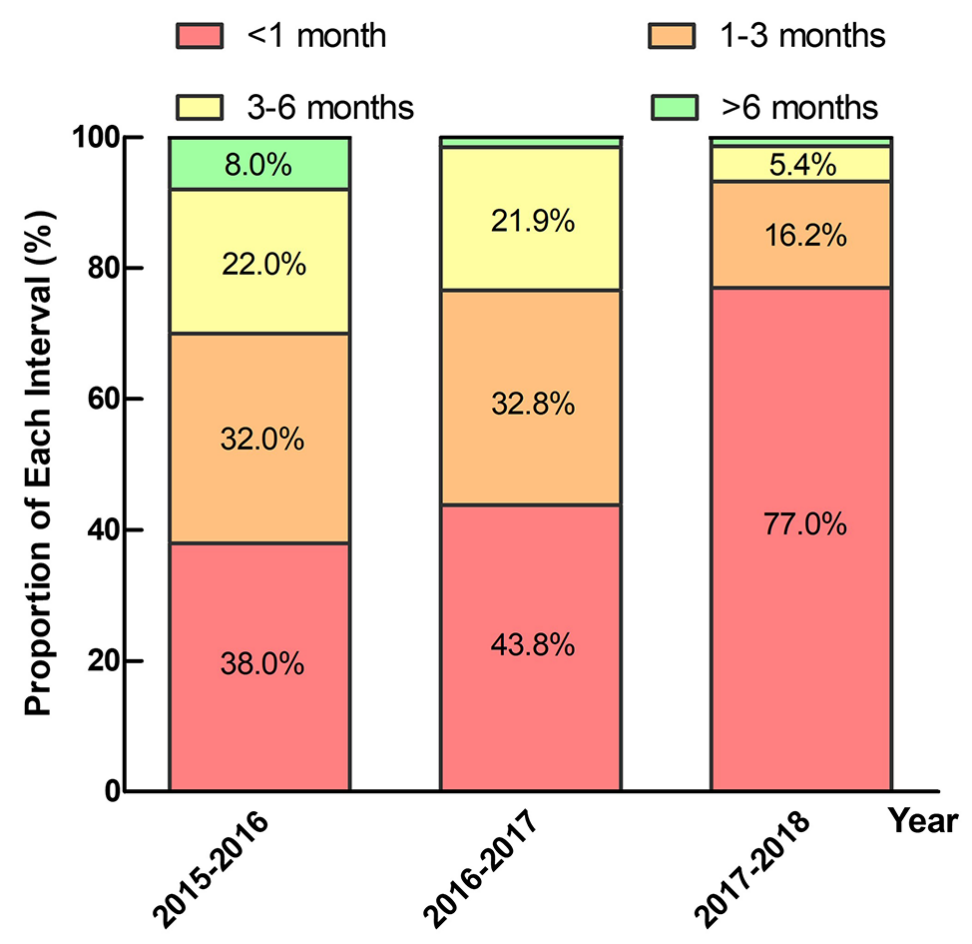
Proportion of each PT-INR monitoring interval based on the year of mechanical heart valve replacement. The proportion of frequent PT-INR monitoring was 38.0%, 43.8% and 77.0%, respectively based on the year of MHV replacement from 2015 to 2018, with a significant increasing trend (*P* < 0.01)

The median follow-up duration of all 188 patients was 3.6 years (IQR: 2.6 to 4.4 years). All these patients acknowledged that they must take warfarin lifelong. Figure 3 shows that the proportion of frequent PT-INR monitoring was 38.0%, 43.8% and 77.0%, respectively based on the year of MHV replacement from 2015 to 2018, with a significant increasing trend (*P* < 0.01). A total of 104 (55.3%) patients were classified into the FM group, and 84 (44.7%) patients were classified into the LFM group.

In the FM group, all-cause death occurred in 1 patient, ischemic stroke in 0 patients, and major bleeding in 4 patients. In the LFM group, all-cause death occurred in 7 patients, ischemic stroke in 6 patients, and major bleeding in 3 patients. The main cause of death in the LFM group was major bleeding, mainly intracranial hemorrhage, and recurrent heart failure. The only death in the FM group was a complicated case: a 32-year-old male who had a history of kidney transplantation and received regular hemodialysis due to the deterioration of renal function. He underwent aortic valve replacement after diagnosed with infectious endocarditis. After the MHV replacement, he lived on with hemodialysis until death caused by intracranial hemorrhage. Characteristics of patients with ischemic stroke, major bleeding, or all-cause death occurred during follow-up are summarized in Additional File Table 1.

**Fig. 4:**
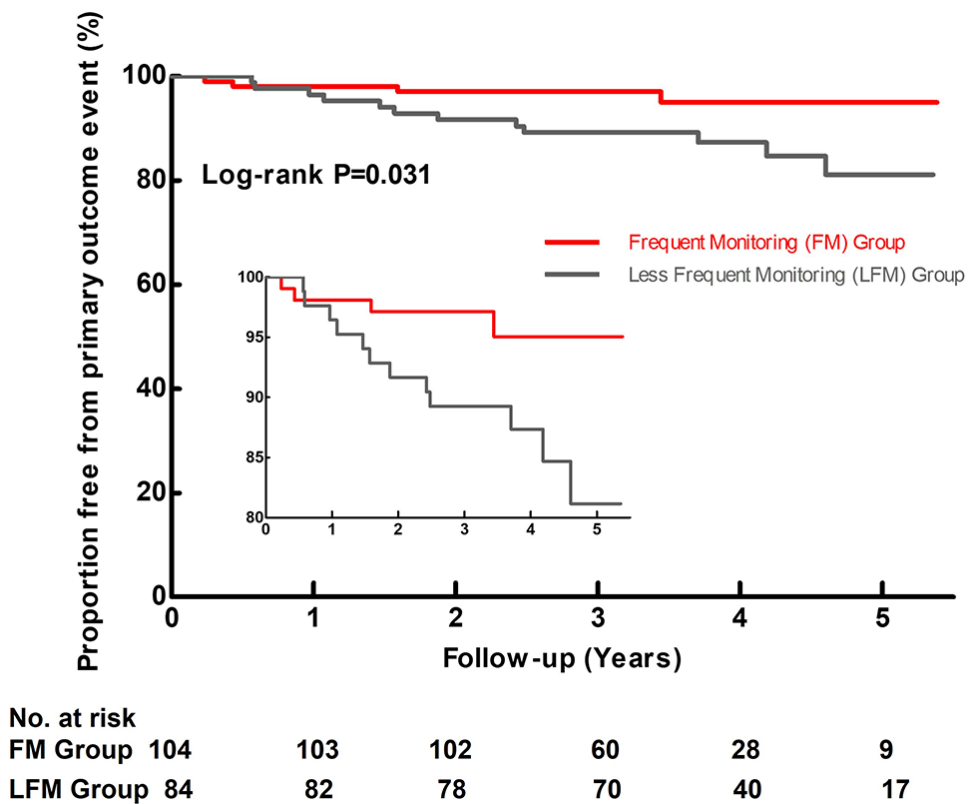
Proportion free from primary outcome event. The Kaplan-Meier curves of primary outcome event free survival in the frequent monitoring group and less frequent monitoring group

The incidences of primary and secondary clinical outcome in the LFM and FM group were shown in Table 2. Based on multivariable Cox analysis, the LFM group remained to have a significantly higher incidence of the primary endpoint event (3.74 vs. 1.16 per 100 patient-years, HR: 3.31 [95% CI 1.05–10.42, P = 0.041]) and all-cause death (2.18 vs. 0.29 per 100 patient-years, HR: 8.33 [95% CI 1.01–66.67, P = 0.049]) after adjustment of age and sex. Figure 4 shows the Kaplan-Meier curves of primary outcome event free survival in the LFM and FM group.

**Table 2:** The incidence of endpoints in FM and LFM group

	Events per 100 patient-years (Number of events)		Unadjusted		Adjusted*	
	LFM Group	FM Group	HR (95% CI)	P	HR (95% CI)	P
Primary endpoints	3.74(12)	1.16(4)	3.24(1.04–10.10)	0.043	3.31(1.05–10.42)	0.041
All cause death	2.18 (7)	0.29 (1)	8.55 (1.04–71.43)	0.045	8.33 (1.01–66.67)	0.049
Ischemic stroke	1.87 (6)	0 (0)				
Major bleeding	0.93 (3)	1.16 (4)	0.87 (0.19–3.90)	0.855	0.95 (0.21–4.37)	0.950

*LFM*: less frequent monitoring; *FM*: frequent monitoring

*With adjustment of age and gender

## Discussion

This telephone-based questionnaire survey demonstrated that the frequency of PT-INR monitoring was clinically unsatisfactory, with only 55.3% of the patients having a monitoring interval of less than 1 month. In addition, patients with less frequent monitoring of PT-INR had a poorer prognosis than those with frequent monitoring.

Patients with MHV replacement are recommended to have their PT-INR values monitored at least once a month [16, 17]. Therefore, we defined frequent monitoring as having a monitoring interval ≤ 1 month. Based on our study, less frequent monitoring of PT-INR was significantly associated with increased risk of the primary composite endpoints and all-cause deaths independent of age and gender. The risk of ischemic stroke was also significantly increased when PT-INR was less frequently monitored. There was no significant difference in the incidence of major bleeding between the FM and LFM groups. However, the death rate due to major bleeding was as high as 50% in our study. Therefore, frequent PT-INR monitoring and appropriate warfarin dose adjustment are crucial for better clinical prognosis in patients after MHV replacement. In some complicated cases, we propose that the PT-INR monitoring interval ≤ 1 month may not be frequent enough. Notably, nearly 75% of the patients in our study had at least one patient-related risk factor (mitral or tricuspid valve replacement, previous thromboembolism, atrial fibrillation, mitral stenosis of any degree, or left ventricular ejection fraction < 35%) listed in the 2021 ESC/EACTS guidelines [18]. We speculate that frequent monitoring of PT-INR not only ensures timely adjustment of warfarin dose but also optimizes other medical treatments and improves the general conditions of the patients.

In our study, the overall proportion of patients with frequent monitoring was 55.3%, and it decreased significantly as postoperative time was prolonged. This reflected that patient compliance was becoming worse over time. In patients with MHV replacement, medical compliance has a particularly large impact on their outcomes after being discharged [19, 20]. The reasons for patients having a less frequent monitoring of PT-INR might be the following: (1) Low spatial accessibility of health services: many patients live in remote regions away from hospitals, which makes it inconvenient for them to have PT-INR tests; (2) Insufficient patient awareness: patients may not truly understand the importance of frequent monitoring of PT-INR and dose adjustment of warfarin, or their level of attention might decline over time. In recent years, advances in technology have made it possible for patients to reach their doctors through smartphone social media apps or telemedicine platforms. However, in the past, patients heavily relied on telephone to contact their doctors. Developing a closer relationship with their doctors may positively impact patients’ compliance. Telemonitoring has been widely used in clinical practices for follow-up [21, 22]. Internet-based management could be an alternative for patients in remote regions. Zhu et al. reported that internet-based warfarin management was superior to the conventional method, as it could reduce anticoagulation complications in patients who received long-term warfarin treatment after MHV replacement [23]. To improve the quality of warfarin therapy, we proposed the following suggestions: (1) Intensify patient education: to emphasize the importance of frequent monitoring of PT-INR and appropriate warfarin dose adjustment; (2) Encourage the use of home-based self-monitoring of PT-INR and internet-based management of warfarin dose adjustment, especially for patients living in remote regions (3) Improve the capacity of long-term postoperative management for patients with MHV replacement by primary healthcare providers.

In our study, most of the baseline characteristics were balanced between patients with follow-up and patients who were lost to follow-up. Therefore, the impact of lost to follow-up on the conclusions might not be significant. However, the high rate of lost to follow-up in patients of 2015–2017 does reflect the shortcomings of the current follow-up system, which requires the joint efforts of both doctors and patients to improve it.

To the best of our knowledge, this is the first study to investigate warfarin monitoring frequency and its correlation with clinical outcomes after MHV replacement in Mainland China. However, there are some limitations to the study: (1) This study was a telephone-based questionnaire survey, so it was not feasible to obtain all the PT-INR values, which made it impossible to compare the difference in PT-INR levels between the 2 groups. Theoretically, frequent testing can ensure timely adjustment of warfarin dose to achieve a higher level of TTR and a better clinical outcome. (2) The sample size of this study is small. Whether the conclusion can be generalized to a larger sample size needs to be further verified. Prospective cohort studies or even randomized controlled studies that allow detailed recording of each PT-INR detection during the study are more convincing. In addition, we were unable to obtain statistically significant results regarding the relationship between anticoagulation intensity and embolic risk in patients with different locations of replaced valves due to limited statistical power. (3) Although we attempted every available method to reach out to the patients, the rate of lost to follow-up was unfortunately high. It indicates the unfavorable real-world conditions in our center. Clearly, this serves as a wake-up call for us to enhance our efforts in monitoring patients post-discharge to reduce the rate of lost to follow-up. (4) It remains uncertain that the use of one month as a criterion to determine whether the PT-INR is monitored frequently is ready for widespread implementation, as its feasibility may be affected by factors such as the level of economic development, proximity to hospitals, and other relevant considerations. (5) In addition, the effect of CYP2C9 and VKORC1 gene polymorphisms on the metabolism of warfarin should also be considered in future studies [24, 25].

## Conclusion

The management of warfarin treatment in patients after MHV replacement remains challenging. Less frequent monitoring of PT-INR might be associated with worse clinical prognosis compared with frequent PT-INR monitoring. Large-scale efforts are urgently needed to reduce warfarin related adverse consequences in patients after MHV replacement in China.

## Electronic supplementary material

Below is the link to the electronic supplementary material.

Additional File 1: Appendix has been provided by the authors to give readers additional information about their work

## Data Availability

The data generated and analyzed during the current study available from the Prof. Mingfang Li on reasonable request.

## List of Abbreviations

BHV: Bioprosthetic heart valve
FM: Frequent monitoring
LFM: Less frequent monitoring
MHV: Mechanical heart valve
PT-INR: Prothrombin time-the international normalized ratio
TTR: Time in therapeutic range
VHD: Valvular heart disease
