# Supplementary material for: Frequency of prothrombin time-international normalized ratio monitoring and the long-term prognosis in patients with mechanical valve replacement
Source: BMC Cardiovasc Disord. 2023 Jun 24;23:322. doi: 10.1186/s12872-023-03293-w (PMC10290782; doi:10.1186/s12872-023-03293-w)
Supplement: Supplementary file 1 — Additional File 1: Appendix has been provided by the authors to give readers additional information about their work [file 12872_2023_3293_MOESM1_ESM.docx]

**Frequency of Prothrombin Time-International Normalized Ratio Monitoring and the Long-term Prognosis in Patients with Mechanical Valve Replacement**

Le Geng^1^*, MD; Jiaxi Gu^1^*, MD; Minghui Li^1^, MD; Hong Liu^1^, MD; Haoliang Sun^1^, MD; Buqing Ni^1^, MD; Weidong Gu^1^, MD; Yongfeng Shao^1^, MD; Mingfang Li^2^, MD, PhD; Minglong Chen, MD

**Supplementary Appendix**

This appendix has been provided by the authors to give readers additional information about their work.

**List of Contents**

Page 1: Cover page

Page 2: List of contents

Page 3: Questionnaire

Page 4: Supplemental Table 1

Page 5: Supplemental Table 2

Page 6: Supplemental Table 3

Page 7: Supplemental Table 4

**Questionnaire on PT-INR monitoring frequency and endpoints of patients with mechanical heart valve replacement**

1. Do you know you should take warfarin for a lifelong time?
2. Did you take warfarin on time every day?
3. How often did you get PT-INR tested?
4. ≤1 month; ②1-3 month; ③ 3-6 month; ④>6 month
5. How is your health status?

(If the patient is still alive, answer question No. 6; if the patient has died, the family member will answer question No.5.)

1. What was the reason of death?

①embolic events; ②major bleeding; ③heart failure; ④malignant tumor; ⑤unexplained reasons; ⑥unknown

1. Have you ever suffered thromboembolic events after operation?

(If yes, answer question No.7; If no, answer question No.8.)

1. Which kind of thromboembolic events have you ever suffered?

①Ischemic stroke; ②TIA; ③Systemic embolism; ④Myocardial infarction; ⑤Thrombus related to valve

1. Have you ever suffered bleeding after operation?

If yes, answer question No.9 and No. 10.

1. The site of bleeding?
2. Intracranial hemorrhage; ②Gastrointestinal bleeding; ③ Urine bleeding; ④Other major bleeding
3. Did you need blood transfusion or did your hemoglobin level decrease for at least 2 g/dl?
   1. Yes; ②No.

**Supplemental Table 1.** Baseline characteristics of patients in follow-up group and lost

to follow-up group.

| Variables | Follow-up (n=188) | Lost to follow-up (n=42) | *P* |
| --- | --- | --- | --- |
| Demographics |  |  |  |
| Male (n, %) | 94 (50%) | 20 (48%) | 0.78 |
| Age (y) | 47.7±7.9 | 47.9±6.8 | 0.87 |
| Disease history |  |  |  |
| Hypertension (n, %) | 42 (22%) | 2 (5%) | 0.01 |
| Diabetes (n, %) | 6 (3%) | 0 (0%) | 0.60 |
| Coronary artery disease (n, %) | 11 (6%) | 1 (2%) | 0.70 |
| Atrial fibrillation (n, %) | 85 (45%) | 22 (52%) | 0.40 |
| Ischemic stroke (n, %) | 24 (13%) | 4 (10%) | 0.79 |
| Creatine clearance (mL/min) | 102.6±33.0 | 97.7±27.2 | 0.43 |
| Cardiac Echo parameters |  |  |  |
| Left atrial diameter (mm) | 47.1±10.3 | 50.6±0.6 | 0.05 |
| Left ventricular diastolic diameter (mm) | 55.2±10.2 | 53.9±10.7 | 0.46 |
| Left ventricular ejection fraction (%) | 60.4±7.1 | 59.6±9.5 | 0.52 |
| Left atrial embolus, n (%) | 20 (11%) | 5(12%) | 0.81 |
| Rheumatic cause (n, %) | 86 (46%) | 29 (69%) | 0.01 |
| Surgery information |  |  |  |
| Mitral valve alone (n, %) | 71 (37.8%) | 12 (28.6%) | 0.29 |
| Aortic valve alone (n, %) | 58 (30.8%) | 7 (16.7%) | 0.09 |
| Mitral + Aortic valve (n, %) | 58 (30.8%) | 20 (47.6%) | 0.05 |
| Concomitant atrial fibrillation ablation (n, %) | 74 (39.4%) | 22 (52.4%) | 0.12 |
| Concomitant CABG (n, %) | 9 (4.8%) | 1 (2.4%) | 0.69 |

Abbreviations: CABG: coronary artery bypass grafting.

**Supplemental Table 2.** Characteristics of patients with all-cause death occurred during follow-up

| **No.** | **Gender** | **Age** | **Monitoring interval ≤1 month** | **Previous stroke history** | **Previous atrial fibrillation** | **Baseline**  **CHA_2_DS_2_-VASc score** | **Concomitant**  **Anti-platelet drugs** | **Cause of death** |
| --- | --- | --- | --- | --- | --- | --- | --- | --- |
| 1 | Female | 51 | No | No | Yes | 1 | No | Ischemic stroke |
| 2 | Male | 52 | No | No | Yes | 0 | Yes | Recurrent heart failure |
| 3 | Female | 58 | No | Yes | Yes | 4 | No | Major bleeding |
| 4 | Male | 43 | No | Yes | No | 3 | No | Major bleeding |
| 5 | Male | 52 | No | No | No | 3 | Yes | Recurrent heart failure |
| 6 | Female | 52 | No | Yes | Yes | 3 | No | Major bleeding |
| 7 | Male | 43 | No | No | No | 0 | No | Recurrent heart failure |
| 8 | Male | 32 | Yes | No | No | 1 | No | Major bleeding |

**Supplemental Table 3.** Characteristics of patients with ischemic stroke occurred during follow-up

| **No.** | **Gender** | **Age** | **Monitoring interval ≤1 month** | **Previous stroke history** | **Previous atrial fibrillation** | **Baseline**  **CHA_2_DS_2_-VASc score** |
| --- | --- | --- | --- | --- | --- | --- |
| 1 | Female | 60 | No | No | Yes | 1 |
| 2 | Female | 51 | No | No | Yes | 0 |
| 3 | Male | 46 | No | No | No | 4 |
| 4 | Female | 52 | No | Yes | Yes | 3 |
| 5 | Female | 45 | No | No | No | 3 |
| 6 | Male | 52 | No | Yes | Yes | 3 |

**Supplemental Table 4.** Characteristics of patients with major bleeding occurred during follow-up

| **No.** | **Gender** | **Age** | **Monitoring interval ≤1 month** | **Bleeding** | **Death after major bleeding** |
| --- | --- | --- | --- | --- | --- |
| 1 | Female | 58 | No | Intracranial hemorrhage | Yes |
| 2 | male | 43 | No | Intracranial hemorrhage | Yes |
| 3 | Female | 52 | No | Intracranial hemorrhage | Yes |
| 4 | male | 32 | Yes | Intracranial hemorrhage | Yes |
| 5 | Female | 52 | Yes | Intracranial hemorrhage | No |
| 6 | Male | 44 | Yes | Gastrointestinal Bleeding | No |
| 7 | Female | 37 | Yes | Gastrointestinal Bleeding | No |
